# Ablation at interatrial connections in biatrial flutter following anteroseptal mitral isthmus line: a case series

**DOI:** 10.1093/ehjcr/ytaf297

**Published:** 2025-06-23

**Authors:** Tadhg Prendiville, Charlie O’Kelly, Louisa O’Neill, David Keane

**Affiliations:** Department of Cardiology, Blackrock Clinic Hospital, Rock Road, Blackrock, Dublin A94E4X7, Ireland; Department of Cardiology, Blackrock Clinic Hospital, Rock Road, Blackrock, Dublin A94E4X7, Ireland; Department of Cardiology, Blackrock Clinic Hospital, Rock Road, Blackrock, Dublin A94E4X7, Ireland; Department of Cardiology, Blackrock Clinic Hospital, Rock Road, Blackrock, Dublin A94E4X7, Ireland; Cardiologist and Electrophysiologist, Director Cardiac Arrhythmia Service, St. Vincent’s University Hospital, Elm Park, Dublin 4 D04T6F4, Ireland

**Keywords:** Biatrial flutter, Catheter ablation, Epicardial conduction, Atrial fibrillation, Case report, Case series

## Abstract

**Background:**

Left atrial perimitral flutter is a common late complication of atrial fibrillation ablation. Repeat ablation with a posterolateral mitral isthmus line to eliminate the flutter circuit has proven technically challenging, leading some to opt for anteroseptal line ablation as an alternative. A significant issue with the latter approach is the emergence of a biatrial flutter using the right atrial septum via epicardial connections to bypass the line of block and allow continued flutter propagation. In this case series we present our experience of biatrial flutter in patients with previous anteroseptal left atrial ablation, and discuss the diagnosis and management of this arrhythmia.

**Case summary:**

We reviewed all cases of biatrial flutter treated at our institution from January 2017 to July 2024. Details of electro-anatomical mapping, ablation strategies and follow-up are described. Four cases of biatrial flutter were identified. All patients were male and had undergone prior pulmonary vein isolation and anteroseptal line. Ablation at sites of interatrial conduction successfully terminated biatrial flutter in 3 out of 4 cases. The remaining case had an unsuccessful ablation and was cardioverted to sinus rhythm. All cases remained free of recurrent atrial arrhythmia at most recent follow-up.

**Discussion:**

Biatrial flutter is a potential complication following anteroseptal line formation for perimitral flutter, necessitating biatrial mapping for diagnosis. Ablation at interatrial connection sites where earliest activation is identified is a potentially effective treatment strategy.

Learning points

- Biatrial flutter can occur following anteroseptal line ablation for perimitral flutter, creating a clockwise or counterclockwise circuit using interatrial connections at Bachmann’s bundle and the coronary sinus.
- Ablation at either interatrial connection is a potential treatment strategy for biatrial flutter, though this may result in delayed left atrial activation in sinus rhythm.

## Introduction

Perimitral left atrial flutter is the one of the most common organized tachyarrhythmias in patients who have undergone pulmonary vein isolation (PVI) for the management of atrial fibrillation (AF). Treatment of perimitral flutter involves creation of a line of block from the mitral annulus to one of the pulmonary veins, i.e. a mitral isthmus (MI) line. This ablation line would typically be performed from the posterolateral mitral annulus to the inferior aspect of the left inferior pulmonary vein antrum; however, thickness of the atrial muscle in this region in some patients and restrictive variations in the coronary sinus and great cardiac vein can render this line difficult, with a low long-term success reported in many series.^1–3^

A left atrial anteroseptal (‘seatbelt’) line which connects the anterior (anteroseptal) mitral annulus to the anterior aspect of the right superior pulmonary vein (RSPV) antrum has been adopted at many centres as an alternative strategy to creation of a conventional posterolateral MI line. The anteroseptal mitral line deliberately avoids connection to a left atrial roof line (which is the superior line created when left atrial posterior wall box isolation is performed) as it is recognized that a line connecting the anterior mitral annulus to a left atrial roof line causes block of Bachmann’s bundle (BB) conduction and as a result delayed contraction of the left atrial appendage with potential stroke risk even when sinus rhythm is maintained^4^.

Once a left atrial anteroseptal line with conduction block has been achieved a mitral annular flutter limited to the left atrium is terminated. However, in some patients a longer flutter circuit can emerge which utilizes both right and left atria. Involvement of the right atrial septum or tricuspid annulus as part of the circuit has been described.^5^

We present a case series of biatrial flutter involving the mitral annulus and septal right atrium (RA) in four patients following anteroseptal MI line ablation.

## Summary figure

**Figure:**
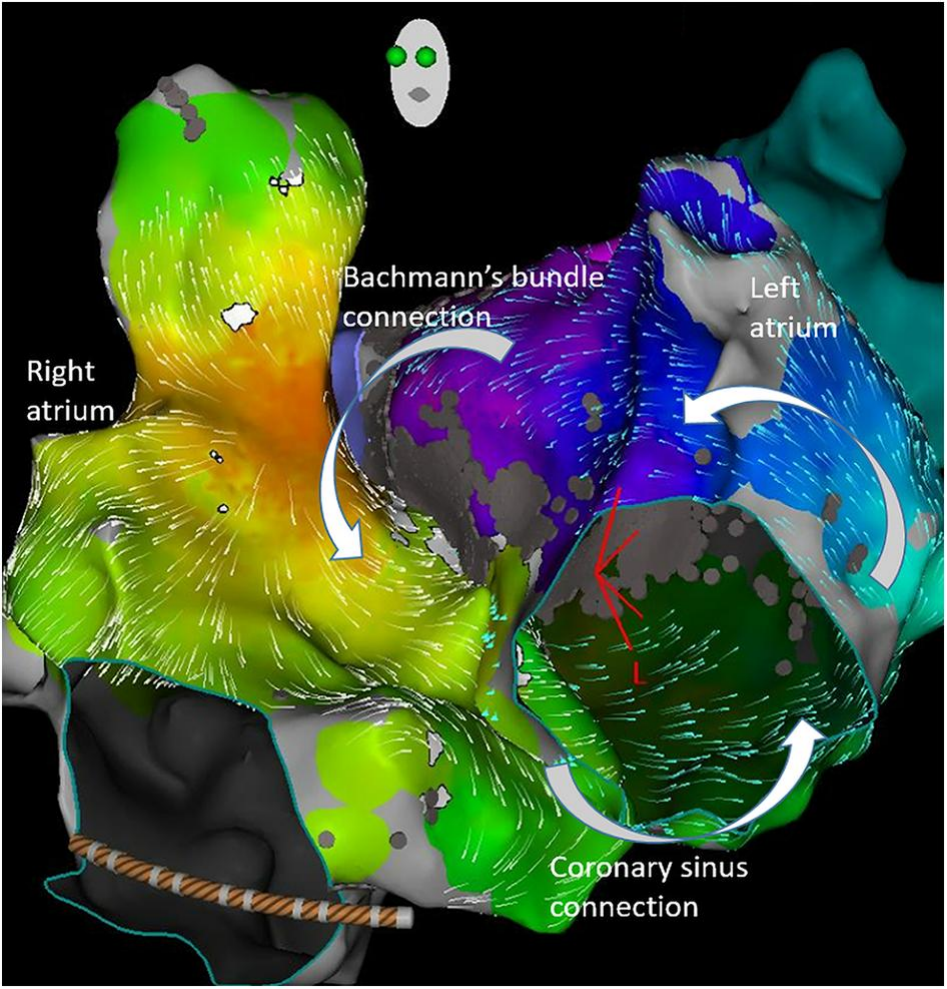


## Procedure

For each case, right femoral venous access (7 and 10-French sheaths) was obtained, with a 20-pole mapping catheter advanced into the coronary sinus (CS). A 3-D electro-anatomical mapping system (Carto, Biosense Webster) was used with pre-procedural CT guidance. Transseptal puncture was performed using an SL-1 transseptal sheath and modified curve Brock-0 XS needle. An Agilis deflectable sheath (Abbott Cardiovascular) was then introduced into the left atrium (LA), with activation mapping performed using a multielectrode eight-splined catheter (Octaray, Biosense Webster). Entrainment mapping and radiofrequency ablation (RFA) were performed via a Q-Dot ablation catheter (Biosense Webster) with a post-pacing interval minus tachycardia cycle length (PPI-TCL) of <30 ms used to determine if points entrained were within the reentrant circuit.

## Case 1

A 58-year-old male presented with recurrent atrial flutter 5 years following a PVI, LA posterior wall box isolation and anteroseptal line ablation, which was performed for persistent AF. Activation mapping revealed a clockwise perimitral flutter with tachycardia cycle length (TCL) of 217 ms. Ablation lines at the left pulmonary vein carina and posterior wall box (adjacent to its connection to the RSPV) were reinforced, with no effect on the tachycardia. A gap in the anteroseptal line was then ablated, with slight prolongation of the TCL to 230 ms. Re-mapping of the LA showed epicardial breakthrough to the anterior LA wall adjacent to the anteroseptal line, with ongoing flutter. A posterolateral MI line was then created, which did not terminate the tachycardia.

**Figure 1:**
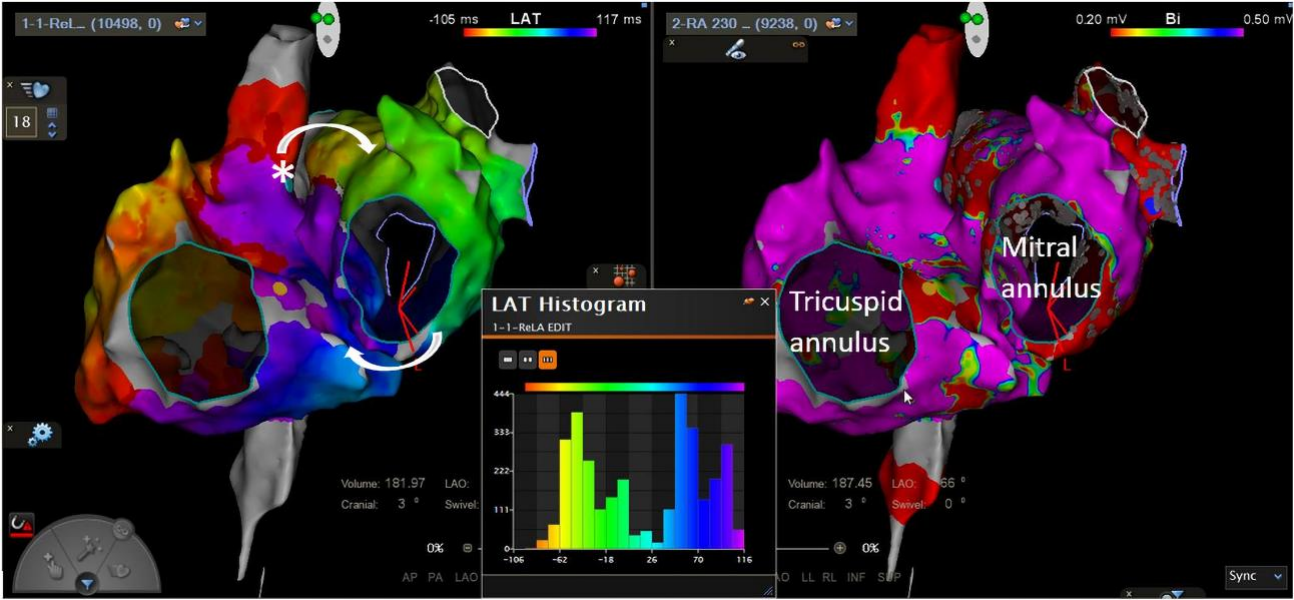
Biatrial activation map (left) and voltage map (right) in LAO view with left atrial time window histogram (centre) showing a missing portion of the TCL, which was mapped to the high septal RA (marked with *). Epicardial connections via BB (superior) and CS (inferior) are marked with curved arrows. BB, Bachmann’s bundle, CS, coronary sinus; LAO, left anterior oblique; RA, right atrium.

**Figure 2:**
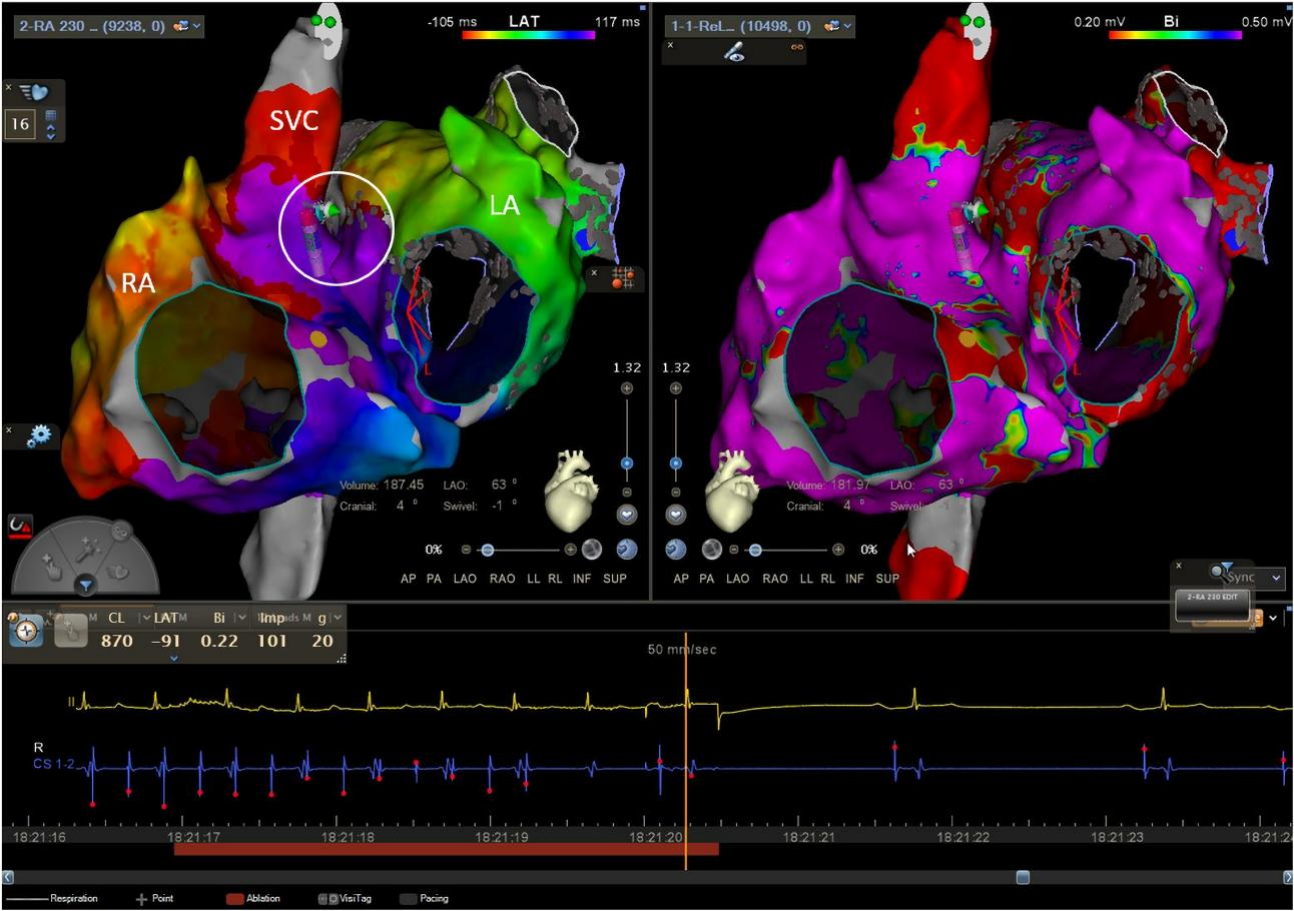
Ablation at the superior cavoatrial junction (circled) terminates the tachycardia. LA, left atrium; RA, right atrium; SVC, superior vena cava.

Further activation mapping showed a missing portion of the time window histogram (*Figure 1*), which was then mapped to the high septal RA. Entrainment at the mid and high RA septum revealed a PPI-TCL of 32 and 13 ms respectively, indicating that the first was very close to the reentrant circuit and the second was within the circuit. RFA at the high RA septum and superior cavoatrial junction subsequently terminated the flutter (*Figure 2*). Sinus rhythm was maintained at the patient’s most recent follow-up (12 months post-procedure).

## Case 2

**Figure 3:**
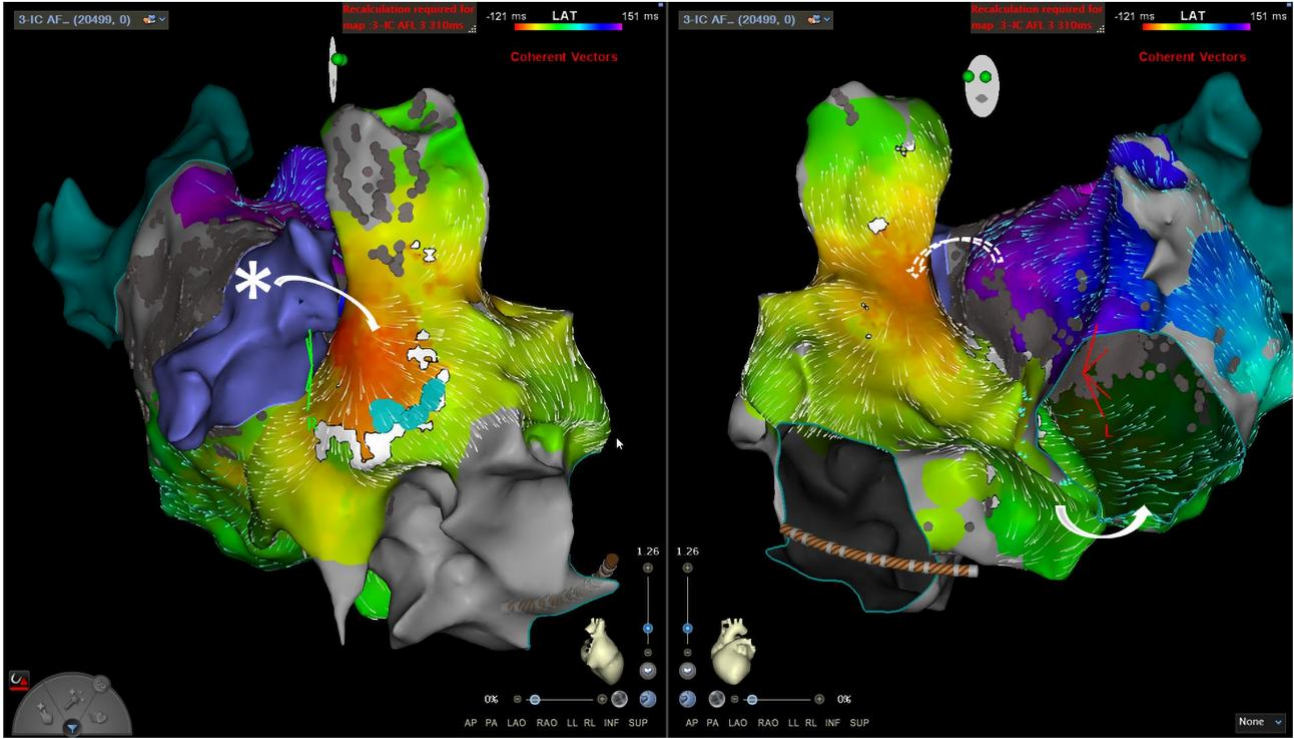
Biatrial activation map in right lateral and LAO views showing counterclockwise biatrial flutter, with conduction from the RSPV antrum (marked with *) to the posterior high right atrium (red area). Epicardial connections are marked with curved arrows. LAO, left anterior oblique.

**Figure 4:**
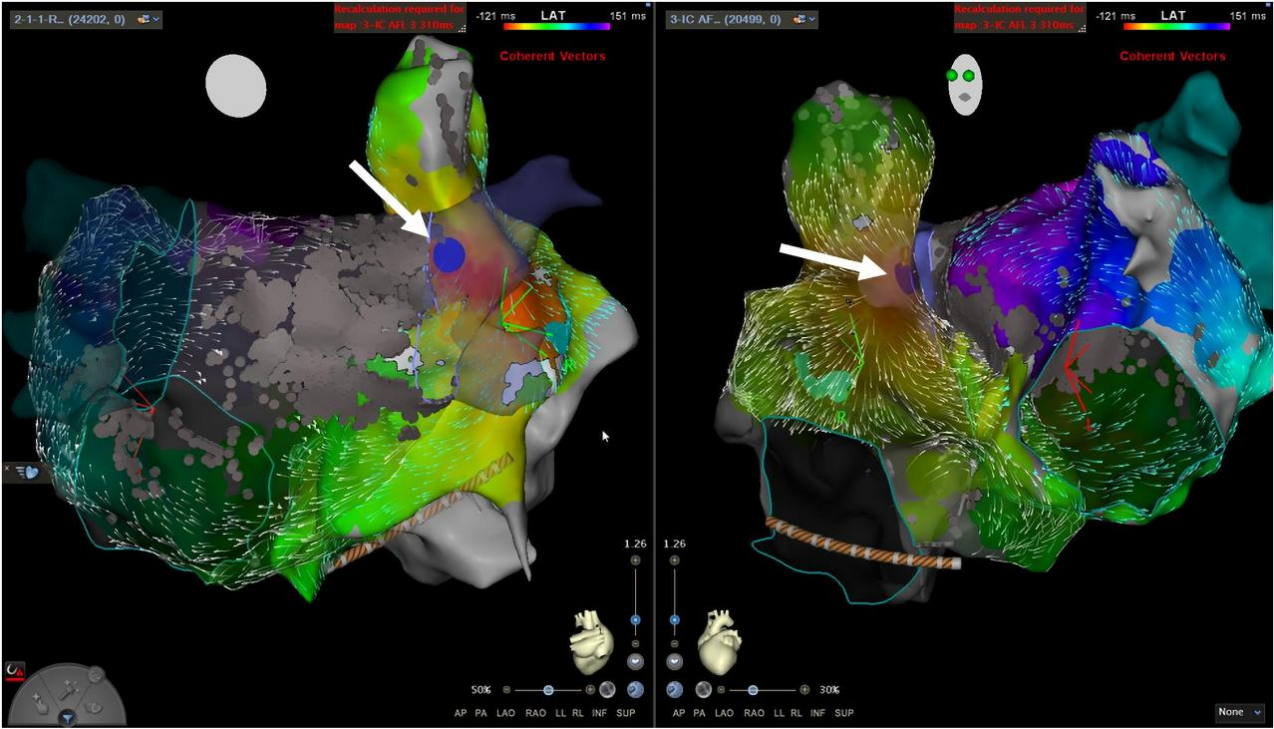
PA and LAO angles of the ablation site at the high posterior right atrium (blue dot marked with arrows) which terminated the tachycardia. LAO, left anterior oblique; PA, posteroanterior.

A 69-year-old male with symptomatic atrial flutter underwent a repeat electrophysiology study. He had previously undergone PVI with a LA roof line for persistent AF, and subsequently a LA posterior wall box isolation, anteroseptal line and cavotricuspid isthmus (CTI) ablation for recurrent AF and flutter. LA mapping revealed a clockwise perimitral flutter (TCL 320–380 ms) with no gap in the prior anteroseptal line but with epicardial bridging via BB. Entrainment from the LA converted the flutter to sinus rhythm, and a posterolateral MI line was performed (with bidirectional block achieved). Following this a counter-clockwise atrial flutter emerged (TCL 312 ms) with involvement of the RA (*Figure 3*). Entrainment in the RA showed the lateral wall, inferior wall and superior cavoatrial junction were not within the reentrant circuit (PPI-TCL all >100 ms) but earliest activation was noted in the posterior high RA (consistent with conduction from the anterior aspect of the RSPV antrum). RFA at this point first slowed and then terminated the tachycardia (*Figure 4*). At 12-month follow-up he remained free of atrial arrhythmia.

## Case 3

A 75-year-old male with a background of hypertrophic cardiomyopathy and prior right atrial CTI ablation underwent an electrophysiology study for recurrent atrial tachycardia (AT). RA mapping following induction of AT showed two arrhythmias—one with earliest activation at the septal SVC (TCL 330 ms) which then changed to a second AT with earliest activation lower on the septum (TCL 290 ms). Both showed activation from proximal to distal on the CS catheter. Transseptal puncture was performed, with voltage mapping in the LA showing patchy areas of low voltage on the posterior wall as well as a large area of anterior scar. Activation mapping of the second AT revealed a perimitral flutter. Ablation was then performed from the anterior mitral annulus to a septal area of scar, with termination of the tachycardia.

**Figure 5:**
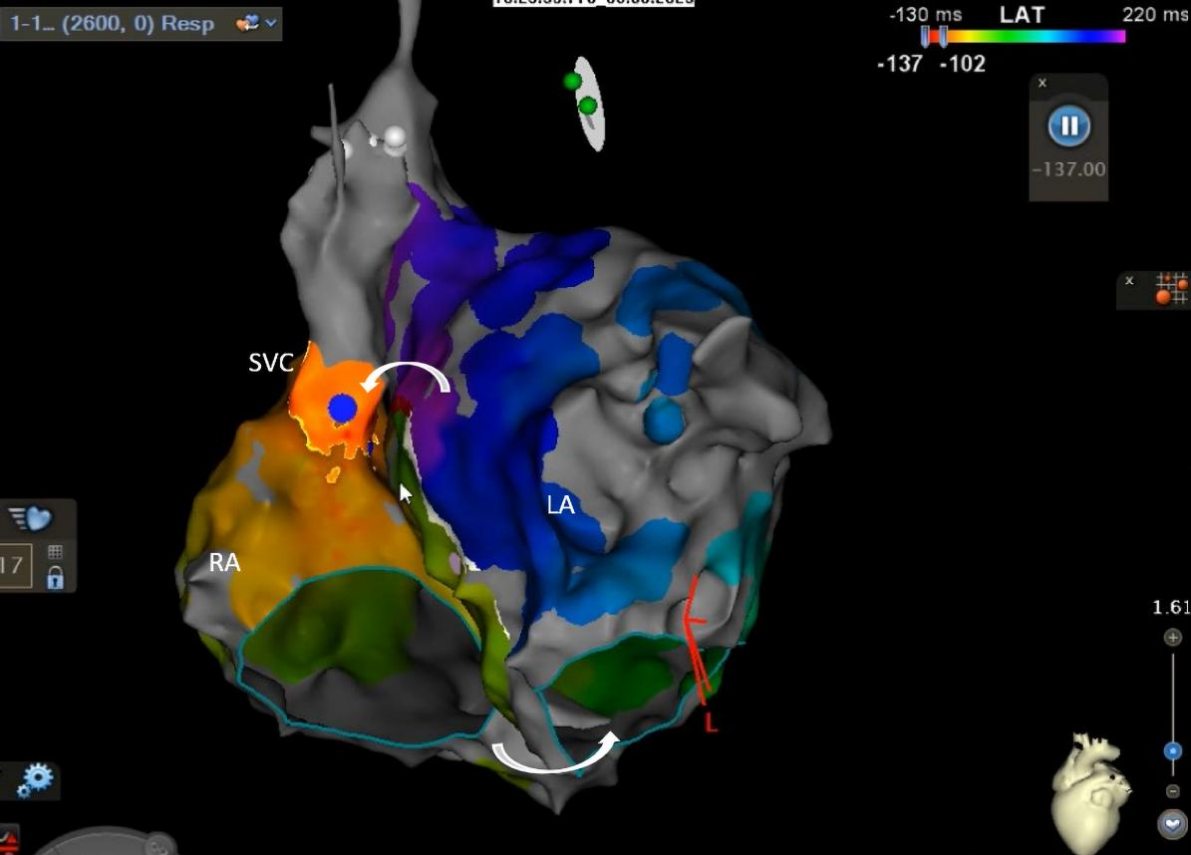
Incomplete biatrial activation map in LAO cranial view showing counterclockwise biatrial flutter with earliest RA activation at the posteroseptal SVC (blue dot). Epicardial connections are marked with curved arrows. LA, left atrium; LAO, left anterior oblique; RA, right atrium; SVC, superior vena cava.

**Figure 6:**
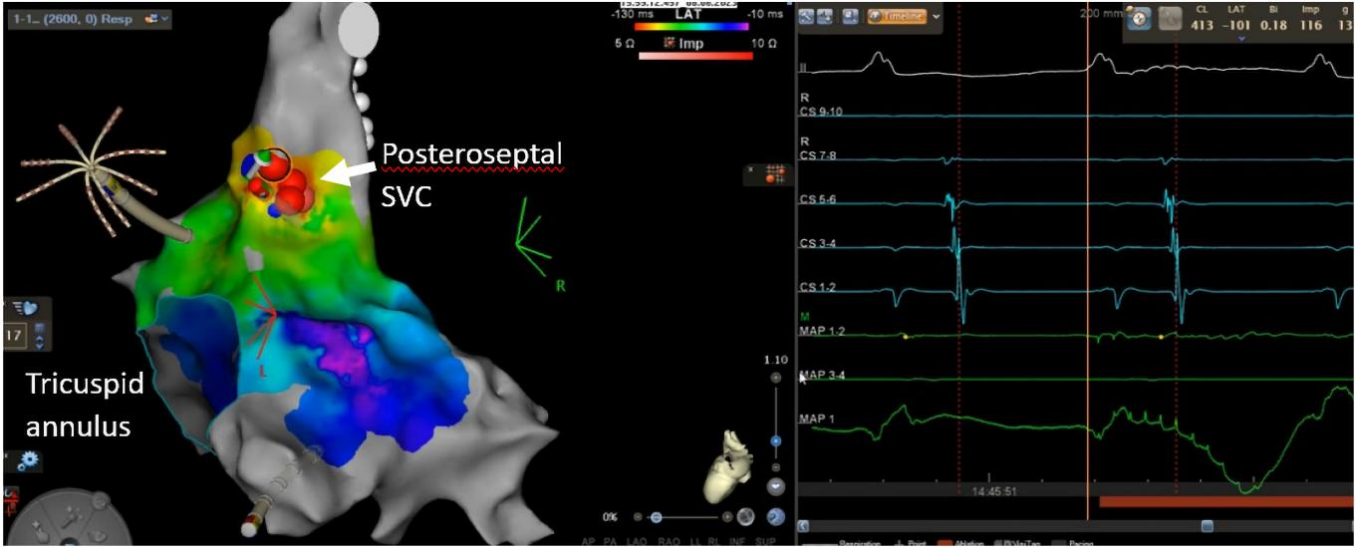
RA activation map in left posterolateral view showing ablation at the posteroseptal SVC which terminated the tachycardia. Ablation lesions are marked by red circles. RA, right atrium; SVC, superior vena cava.

Following confirmation of block across the anteroseptal line, further rapid atrial pacing was performed, which induced a further AT (TCL 390 ms). Repeat biatrial mapping was consistent with a biatrial flutter (*Figure 5*), with earliest activation at the posteroseptal SVC (similar to the first AT)—focal ablation in this area restored sinus rhythm (*Figure 6*), with no inducible arrhythmia on subsequent pacing. At 6 months of follow-up his arrhythmia had not recurred.

## Case 4

**Figure 7:**
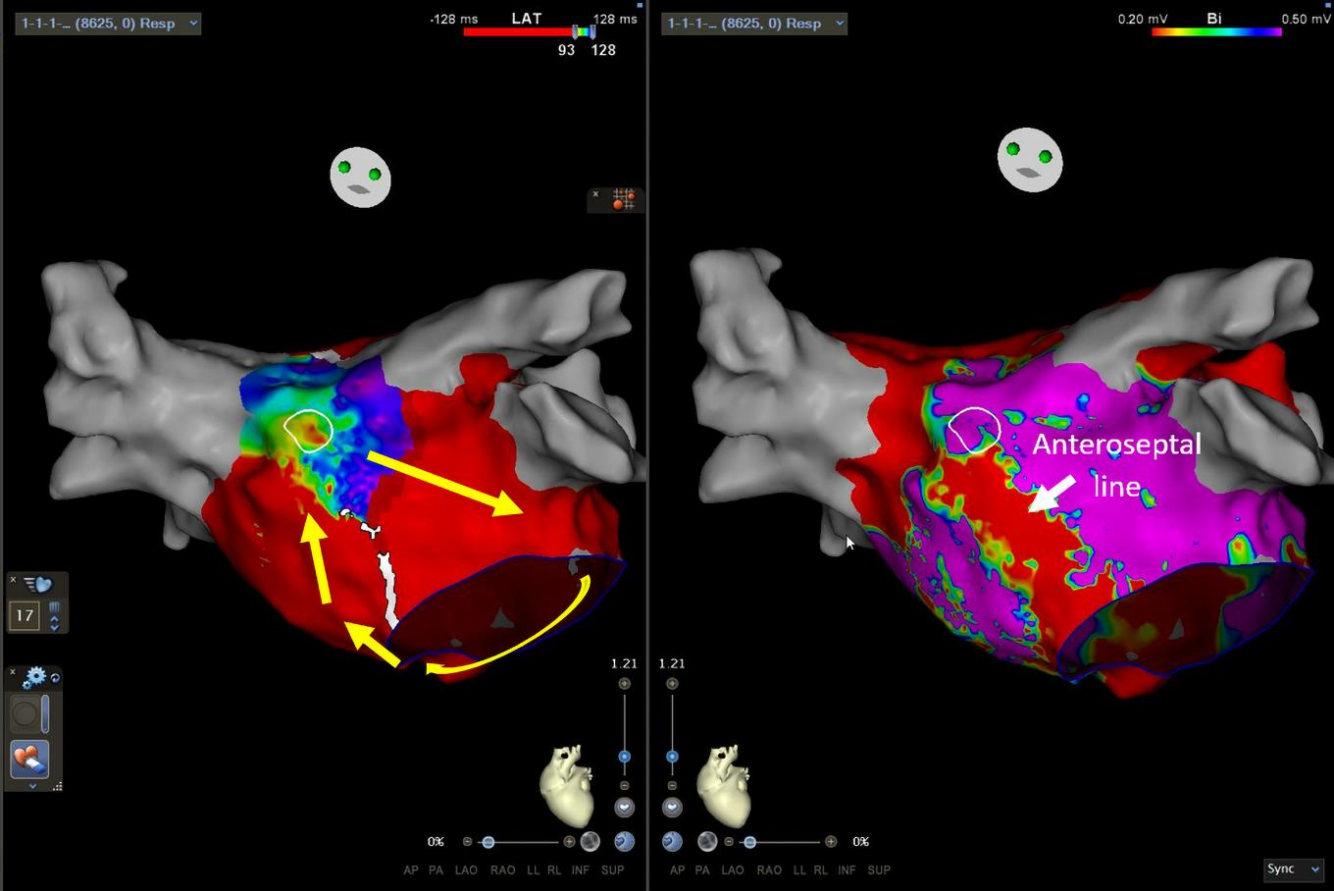
LA activation map (left) and voltage map (right) in cranial view showing epicardial conduction across the anteroseptal line. The direction of LA activation is marked with yellow arrows. Conduction travels from the inferior septum to the line, then is seen to break out from the insertion site of BB on the superior side (circled) without travelling across the line. BB, Bachmann’s bundle; LA, left atrium.

**Figure 8:**
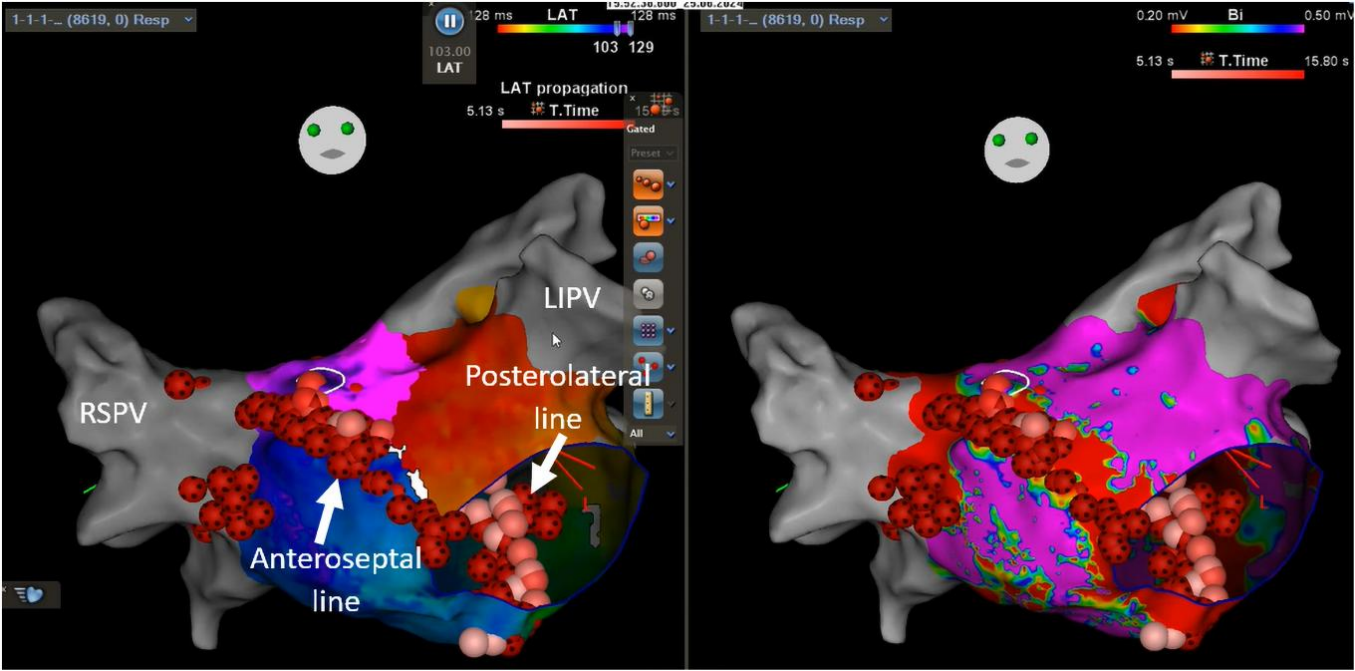
LA activation (left) and voltage (right) maps showing the location of ablation lesions (red and pink circles) along the anteroseptal and posterolateral MI lines, at the right pulmonary vein carina and close to BB insertion. BB, Bachmann’s bundle; LA, left atrium; LIPV, left inferior pulmonary vein; MI, mitral isthmus; RSPV, right superior pulmonary vein.

A 70-year-old male underwent an electrophysiology study for atypical atrial flutter on a background of PVI 6 months previously for paroxysmal AF. Small segments of reconduction at the right pulmonary vein carina were first reisolated, following which a clockwise perimitral flutter was induced by burst pacing (TCL 249 ms). Left atrial anteroseptal line ablation was performed with bidirectional block achieved and termination of the flutter, but a slower flutter then emerged (TCL 280 ms) with biatrial involvement confirmed on entrainment at the RA septum. Ablation at the site of earliest LA activation (close to BB insertion) did not affect the flutter (*Figures 7* and *8*). Formation of a new posterolateral MI line similarly did not terminate the arrhythmia (despite elimination of electrograms through this corridor), and the patient was cardioverted to sinus rhythm. No arrhythmia recurrence was detected at 6-month follow-up.

## Discussion

This case series demonstrates four examples of biatrial flutter due to a macro-reentrant circuit involving the LA and septal RA following left atrial anteroseptal line ablation for perimitral flutter. Clinical and procedural characteristics of these four cases are listed in the Supplementary material online, *Table S1*. This form of atypical flutter occurs as the perimitral impulse returns to the RA via an interatrial connection (IAC) and back to the LA via another IAC. Common IACs include BB, the coronary sinus and the fossa ovalis,^6^ though cases involving the great cardiac vein, septopulmonary bundle and Ligament of Marshall (LOM) have also been described.^7^ Three types of biatrial flutter have been described: Type 1 involving the mitral and tricuspid annuli, Type 2 involving the mitral annulus and RA septum, and Type 3 involving LA septum and RA septum.^5^ Each of our cases represents the second type.

While generally considered uncommon, one series reported biatrial flutter in 4 out of 15 patients (31%) who underwent anteroseptal ablation.^8^ Ablating from the left septum does not appear to penetrate through to the right atrial side thus allowing propagation of the flutter via this aspect of the septum.^8^ Slowed superior interatrial conduction via BB following anteroseptal line with unaffected inferior conduction via the CS may also facilitate reentry.

Atypical atrial flutters can often (though not always) be differentiated from CTI-dependent flutter by the absence of inferior sawtooth waves on 12-lead electrocardiogram,^9^ but require invasive electroanatomic mapping for further localisation (and to differentiate from focal AT). Both activation mapping (showing the sequence of atrial depolarisation) and entrainment mapping (pacing from different points in the atria to determine if they are part of the arrhythmia circuit) are useful in this scenario.

Clues to the presence of biatrial flutter include TCL prolongation without arrhythmia termination following anteroseptal line formation (implying a larger circuit) or an incomplete time window histogram on activation mapping of the LA (implying that part of the circuit is outside the LA, i.e. in the RA or epicardial). Changes in activation may be minimal where mapping is limited to the left atrial endocardium if the interatrial septum remains part of the circuit (but with involvement of the RA aspect rather than LA aspect of the septum as in our cases); therefore, biatrial mapping should be performed where either of the aforementioned clues are present. Entrainment mapping can prove involvement of the RA within the circuit (if PPI-TCL is <30 ms), though it must be determined that the activation sequence and TCL are unchanged post-entrainment as rapid pacing in the atrium can activate a different arrhythmia circuit (or terminate the arrhythmia itself). Activation mapping in the RA completes the time window histogram and shows the direction of septal depolarisation (superior or inferior).

Treatment of this tachycardia involves ablation at sites of interatrial conduction (i.e. close to BB or at an additional IAC), or along a perimitral anatomical isthmus (most commonly the posterolateral isthmus). Alcohol injection into the Vein of Marshall has also shown success in cases where the LOM is involved in the flutter circuit. In our series, Cases 1, 2, and 3 had sinus rhythm restored through RFA at IAC sites, while a lateral MI line did not prevent or terminate the tachycardia in Cases 1 and 2. In Case 4 both ablation near BB and along the lateral MI were unsuccessful. Other sites which could potentially have been targeted in this case include the LOM (as the circuit may have been bypassing the lateral line via this route) or in the CS (though ablation near multiple IACs would carry a high risk of LA conduction delay).

A recent systematic review by Lai *et al*.^7^ reported ablation near IAC sites was more frequently used compared with isthmus ablation, though recurrence rates were similar with both strategies.^7^ Impairment of normal interatrial conduction is a concern with IAC modification, with some degree of altered atrial physiology reported in 40% of cases using this ablation target.^7^ Electrical isolation of the left atrial appendage may result in increased rates of thromboembolism. Furthermore, tachycardia recurrence may occur via another IAC. As such, anatomical posterolateral MI ablation has been recommended as the preferred strategy where feasible,^7^ though this was not borne out in our series.

## Conclusion

Biatrial flutter is a potential sequela following anteroseptal ablation for perimitral flutter. Activation and entrainment mapping can be used to highlight RA involvement, with ablation at the entry or exit site resulting in tachycardia termination in some cases. However, due to the large footprint or ‘landing area’ of BB insertion over the roof of the right and left atria and the depth of such epicardial connections, ablation in such cases can be challenging.

## Lead author biography


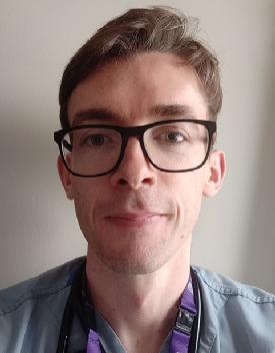


Dr. Tadhg Prendiville completed his medical degree at University College Dublin in 2016, following which he completed internal medicine at Mater Misericordiae University Hospital, Dublin and general cardiology training under the Irish Higher Specialist Training Programme in Cardiology. He is currently pursuing a clinical electrophysiology fellowship at St. Michael's Hospital, University of Toronto, Canada. He is a member of the Royal College of Physicians of Ireland, the Heart Rhythm Society and the European Heart Rhythm Assocation.

## Supplementary Material

ytaf297_Supplementary_Data

## Data Availability

Additional data relating to the cases described will be made available upon reasonable request to the authors.

## Supplementary material

**Consent:** Informed consent was obtained from all patients for inclusion in our series and publication of their cases in an anonymised format, in compliance with guidance from the Committee on Publication Ethics.

**Funding:** None declared.
