# Supplementary material for: Ablation at interatrial connections in biatrial flutter following anteroseptal mitral isthmus line: a case series
Source: Eur Heart J Case Rep. 2025 Jun 23;9(7):ytaf297. doi: 10.1093/ehjcr/ytaf297 (PMC12226450; doi:10.1093/ehjcr/ytaf297)
Supplement: ytaf297_Supplementary_Data [file ytaf297_supplementary_data.docx]

| **Case** | **Age** | **Sex** | **Ablation prior to biatrial flutter** | **Interatrial connections involved** | **Ablation site** | **Outcome** |
| --- | --- | --- | --- | --- | --- | --- |
| 1 | 58 | Male | PVI, LAPWI, ASL | BB, CS | Superior RA septum, cavoatrial junction | Successful |
| 2 | 69 | Male | PVI, LARL, LAPWI, ASL, CTI, PLL | BB (posterosuperior branch), CS | High posterior RA | Successful |
| 3 | 75 | Male | CTI, ASL | BB, CS | Posteroseptal SVC | Successful |
| 4 | 70 | Male | PVI, ASL | BB, CS | Superior LA (close to BB insertion), PLL | Unsuccessful (cardioverted) |

PVI = pulmonary vein isolation, LAPWI = left atrial posterior wall isolation, ASL = anteroseptal line, LARL = left atrial roof line, CTI = cavotricuspid isthmus, PLL = posterolateral line, BB = Bachmann’s bundle, CS = coronary sinus, RA = right atrium, SVC = superior vena cava
